# A ROBOTIC SYSTEM FOR HIGH-THROUGHPUT AUTOMATED LIFESPAN ANALYSIS IN C. ELEGANS

**DOI:** 10.1093/geroni/igz038.383

**Published:** 2019-11-08

**Authors:** Ben Blue, Elena Vayndorf, Matt Kaeberlein, Jason Pitt

**Affiliations:** University of Washington, Seattle, Washington, United States

## Abstract

Over the past decade, the identification of potential genetic and pharmacological modifiers of lifespan and age-related pathologies in C. elegans and other model organisms has yielded fruitful leads for follow-up investigation. A major limitation of such studies, however, is that they are often time-consuming and labor-intensive. The advent of affordable high-quality digital cameras, robotics systems, and 3D printers, as well as the decreasing costs of image storage and processing have allowed us to automate data capture and analysis at an unprecedented scale. To this end, our group developed a tool consisting of an unbiased, high-throughput, automated robotic system to perform genetic and pharmacological quantification of lifespan and health measures in C. elegans and related nematode species. The WormBot utilizes industry-standard, commercially available robotics components to position a digital camera over individual wells of standard 12-well culture plates, containing a small population of C. elegans per well. A high-resolution image is captured of each plate every 10 minutes throughout the course of the experiment. Our software processes the images for stabilization, compiles them into a time-lapse series for each well, and quantifies survival and mobility (paralysis) with minimal input. In addition, a short video is captured of each well once each day, to allow for quantitative analyses of activity and coordinated movement. We will describe this technology and present applications to screen genetic and pharmacological libraries in aging and age-related disease.

